# N-type organic electrochemical transistors with stability in water

**DOI:** 10.1038/ncomms13066

**Published:** 2016-10-07

**Authors:** Alexander Giovannitti, Christian B. Nielsen, Dan-Tiberiu Sbircea, Sahika Inal, Mary Donahue, Muhammad R. Niazi, David A. Hanifi, Aram Amassian, George G. Malliaras, Jonathan Rivnay, Iain McCulloch

**Affiliations:** 1 Department of Chemistry and Centre for Plastic Electronics, Imperial College London, London SW7 2AZ, UK; 2 Materials Research Institute and School of Biological and Chemical Sciences, Queen Mary University of London, Mile End Road, London E1 4NS, UK; 3 Department of Bioelectronics, École Nationale Supérieure des Mines, CMP-EMSE, MOC Gardanne 13541, France; 4 King Abdullah University of Science and Technology, SPERC, Thuwal 23955-6900, Saudi Arabia; 5 Department of Chemistry, Stanford University, Stanford, California 94305, USA; 6 Palo Alto Research Center, Palo Alto, California 94304, USA

## Abstract

Organic electrochemical transistors (OECTs) are receiving significant attention due to their ability to efficiently transduce biological signals. A major limitation of this technology is that only p-type materials have been reported, which precludes the development of complementary circuits, and limits sensor technologies. Here, we report the first ever n-type OECT, with relatively balanced ambipolar charge transport characteristics based on a polymer that supports both hole and electron transport along its backbone when doped through an aqueous electrolyte and in the presence of oxygen. This new semiconducting polymer is designed specifically to facilitate ion transport and promote electrochemical doping. Stability measurements in water show no degradation when tested for 2 h under continuous cycling. This demonstration opens the possibility to develop complementary circuits based on OECTs and to improve the sophistication of bioelectronic devices.

## Abstract (web summary)

Organic electrochemical transistors transduce ionic to electronic signals in aqueous solutions, holding promise for biological sensing applications. Here, Giovannitti et al. report an ambipolar organic electrochemical transistor, based on a conjugated copolymer, which has a high stability in water.

## Graphical abstract

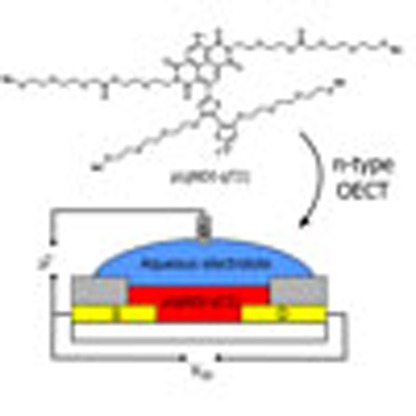

Interest in mixed conduction, as in recent bioelectronic and energy applications, has led to a surge in novel organic electronic materials and devices. One characteristic example is an organic electrochemical transistor (OECT), in which ions from an electrolyte penetrate a polymer film and modulate its conductivity. As a result, OECTs can efficiently transduce ionic signals into electronic ones, making them ideal biological sensing elements. OECTs can be fabricated from biocompatible materials^1^,^2^ and operate in aqueous environments, which enables recordings *in vivo* and *in vitro*^1^,^3^,^4^,^5^. The advantage of OECT-based sensors compared to organic field-effect transistor (OFET)-based sensors is that ions in the former interact with the whole volume of the active material, giving rise to lower impedance and higher transconductance^6^. As a result, the performance of an OECT, defined by the efficiency with which it transduces a voltage modulation at the gate (*V*_G_) into a current modulation in the channel (*I*_D_), depends on the thickness of the active layer. This is contrary to OFETs, which rely on the interfacial accumulation of charges and are thus limited by the double layer capacitance^5^,^6^. In particular, the transconductance (*g*_m_=∂*I*_D_/∂*V*_G_) of OECTs operated in an aqueous environment can reach more than 3.0 mS (refs 3, 7, 8) at low biases, enabling their use in clinical neuroscience applications, including electrocardiography^3^,^9^, electroencephalography^6^,^9^ or neural stimulation^4^.

The current state-of-the-art active material for OECTs is the conducting polymer blend poly(3,4-ethylenedioxythiophene):poly(styrenesulfonate) PEDOT:PSS, where the material is doped in its native state, requiring the OECT to be operated in depletion mode^3^,^8^. The operation of an OECT in accumulation mode allows for low power consumption devices with high ON/OFF ratios. The ability of a polythiophene-based polyelectrolyte to operate as an accumulation mode OECT has previously been demonstrated with transconductances of up to 2.0 mS (ref. 10). Intermediate materials, where conducting and semiconducting polymers were blended together, have also been reported^11^. To date, however, all reported OECTs have relied on hole transport (p-type), while development of electron transporting (n-type) or ambipolar OECTs has been ignored. Such OECTs would allow the development of complementary circuits and will dramatically improve the sophistication of bioelectronics devices. However, the development of appropriate semiconductor materials is a major challenge: It requires that a material be both stable in an aqueous electrolyte, and that it be reversibly reduced and oxidized within the electrochemical window imparted by that electrolyte. This requires the design of materials that concurrently have a high electron affinity (EA), a low ionization potential and the capacity for facile ion penetration. In addition to their ability to be oxidized and reduced efficiently, these materials should also show high electron and hole mobilities in order to sustain large electronic currents and yield efficient current modulation in an OECT format. Recently, the electron mobility of n-type polymers has increased rapidly, reaching values of more than 1.0 cm^−2^ V^−1^ s^−1^ in OFETs,^12^,^13^,^14^,^15^,^16^,^17^ thus enabling n-type OECTs operating in accumulation mode. While air stable n-type materials for OFETs have been reported^18^,^19^, these materials usually degrade when operated in water.

In this work, we report the first n-type and ambipolar OECT which operates in water and shows a high stability during pulse measurements over 2 h. This work paves the way for the fabrication of OECT complementary circuits.

## Results

### Materials synthesis

**Figure 1:**
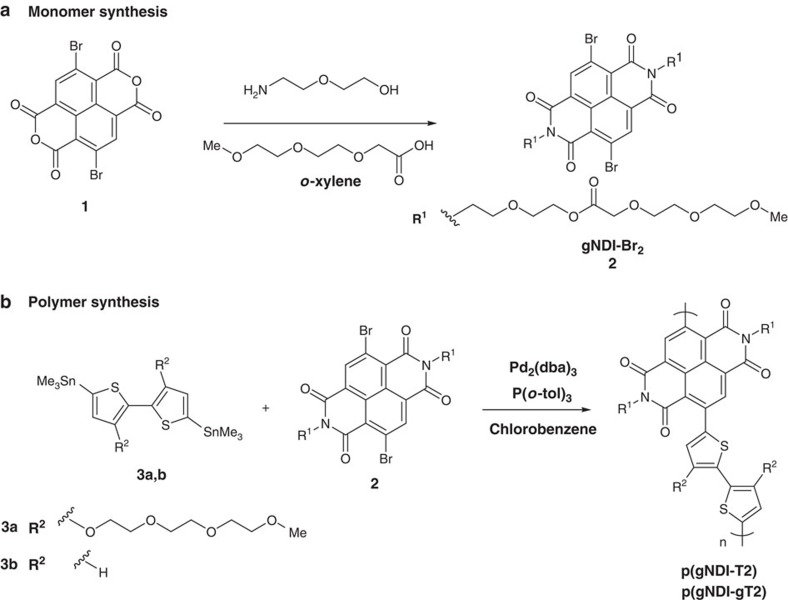
Monomer and polymer synthesis. (**a**) One-pot synthesis of monomer 2 and (**b**) Stille polymerizations affording p(gNDI-T2) and p(gNDI-gT2).

The synthetic design for developing n-type OECT materials required a narrow band gap donor acceptor copolymer with polar side chains. To this end, we focus on the highly electron-deficient 2,6-dibromonaphthalene-1,4,5,8-tetracarboxylic diimide (NDI) monomer which can easily be copolymerized with electron-rich thiophene based co-monomers. The synthesis of linear glycol chains with several ethylene glycol repeating units and an amine end-group which is needed for the imide formation is usually time consuming and involves several reaction steps^20^. Here, we present a simple reaction where the NDI monomer gNDI-Br_2_ (**2**, Fig. 1) with long linear ethylene glycol-based side chains is synthesized in a one-pot reaction from commercial reagents. The amino alcohol forms an ester with 2-[2-(2-methoxyethoxy)ethoxy]acetic acid while the dianhydride **1** is converted to the diimide monomer **2**. Stille polymerization of **2** in chlorobenzene with 5,5′-bis(trimethylstannyl)-2,2′-bithiophene **3b** and the (2-(2-(2-methoxyethoxy)ethoxy)ethoxy) analogue **3a** with 2 mol% Pd_2_(dba)_3_ and P(*o*-tol)_3_ was carried out to synthesize polymers p(gNDI-T2) and p(gNDI-gT2), respectively, following a procedure for semiconducting polymers with polar side chains^21^. The solubility of the polymer in polar solvents increases with the amount of glycol side chains. Polymer p(gNDI-gT2) is soluble in chloroform, 1,1,2,2-tetrachloroethane and dimethylformamide while p(gNDI-T2), with lower glycol side chain density, can only be dissolved in hot 1,1,2,2-tetrachloroethane. End-capping of p(gNDI-gT2) with mono-functionalized thiophenes was performed to remove bromo or organo-tin polymer endgroups. Due to a low solubility of p(gNDI-T2) in chlorobenzene it was not possible to carry out the end-capping procedure. The molecular weight distribution of p(gNDI-gT2) was measured by matrix-assisted laser desorption/ionization time-of-flight spectrometry (MALDI-TOF) where sequences of (gNDI-gT2)_n_ up to seven repeating units (*n*=7) were detected (Supplementary Fig. 16). In addition, ^1^H NMR end-group analysis yielded an average repeating unit of seven repeating units and is in agreement with the MALDI-TOF measurements (Supplementary Fig. 17).

### Electrochemical and optical properties

**Figure 2:**
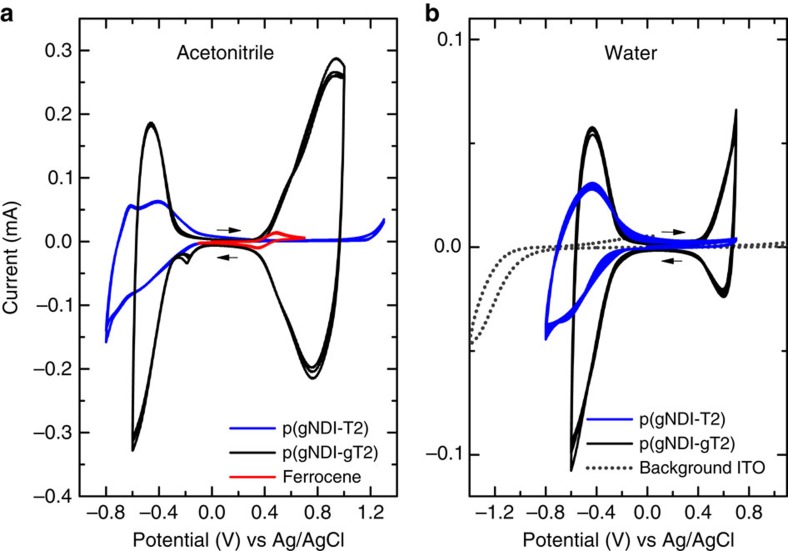
Cyclic voltammetry of thin films in acetonitrile and aqueous electrolyte solutions. The polymers p(gNDI-T2) and p(gNDI-gT2) were deposited by spin coating on ITO-coated glass substrates and measured with a scan rate of 100 mV s^−1^ in (**a**) 0.1 M TBAPF_6_ acetonitrile solution (ferrocene is shown as the reference, three cycles) and (**b**) 0.1 M NaCl aqueous solution (40 cycles) with a background measurement of a neat ITO substrate.

**Figure 3:**
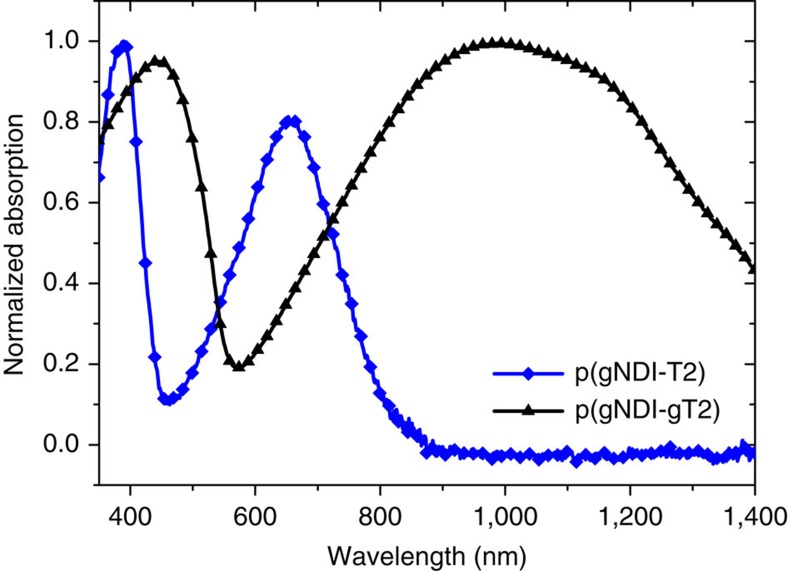
Normalized UV-Vis absorption spectra in solid state. p(gNDI-T2) (blue line) and p(gNDI-gT2) (black line).

Figure 2a presents the cyclic voltammetry (CV) measurements of thin films comprising p(gNDI-T2) and p(gNDI-gT2) on indium tin oxide (ITO)-coated glass substrates in acetonitrile solution. The results of the CV measurements are summarized in Table 1. Polymer p(gNDI-T2) has an electrochemical bandgap of 1.68 eV and polymer p(gNDI-gT2) of 0.71 eV where the substitution in the 3,3′-positions of the bithiophene from a hydrogen atom to a methyl end-capped triethylene glycol chain decreases the ionization potential (IP) from 5.53 to 4.83 eV and increases the EA from 3.85 to 4.12 eV. The thin film UV-Vis-NIR absorption of p(gNDI-T2) and p(gNDI-gT2) is presented in Fig. 3. In the solid state, the absorption onset for polymer p(gNDI-gT2) is at 1,550 nm (Supplementary Fig. 1) and for p(gNDI-T2) at 817 nm which is consistent with an increased donor strength of the glycol-bithiophene unit compared to bithiophene unit and in agreement with literature^22^,^23^. The initial UV-Vis spectrum of p(gNDI-gT2) has two absorption peaks, a *π*-π* transition located between 350 and 550 nm and a broad absorption band from an intramolecular charge transfer complex (ICT) between 600 and 1,550 nm. Time-dependent density functional theory calculations at WB97XD/6-31G(d,p) level of theory were carried out to simulate the UV-Vis spectra of both polymers and compare them to the experimental absorption spectra. A similar trend for the band gap was found where polymer p(gNDI-gT2) shows an ICT transition at lower energies compared to p(gNDI-gT2) in the gas phase (Supplementary Fig. 5). As shown in Fig. 2b, the attachment of polar glycol chains at the polymer backbone enables reversible electrochemical switching between the reduced and neutral states in 0.1 M NaCl aqueous solution for both polymers. During the reduction (n-type doping process), sodium ions drift into the thin film to stabilize the negative charge on the polymer backbone. In the case of the narrower bandgap polymer p(gNDI-gT2), which has a lower ionization potential, oxidation (p-type doping) takes place at relatively low positive voltages where chloride ions are driven into the film to stabilize the positive charge on the backbone. With these synthetic modifications, we are able to achieve stable redox reactions for the conjugated polymers p(gNDI-T2) and p(gNDI-gT2) in 0.1 M NaCl aqueous solution and also in phosphate-buffered saline solution (Supplementary Fig. 11) where the measurements show reversible doping and de-doping over 40 cycles. Remarkably, all CV measurements in aqueous solutions were performed under ambient conditions, without removing oxygen.

**Figure 4:**
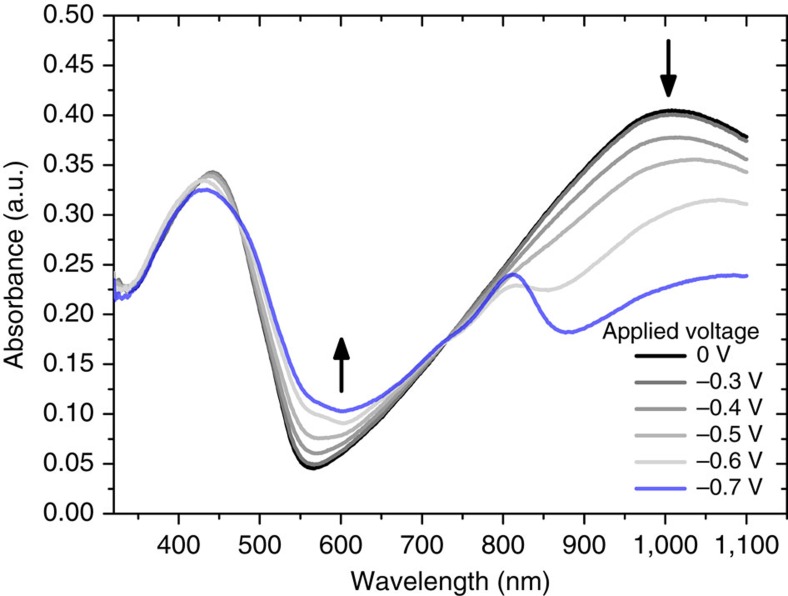
Spectroelectrochemical measurements of p(gNDI-gT2). UV-Vis measurements were carried out while applying a voltage between 0 and –0.7 V versus Ag/AgCl in 0.1 M NaCl aqueous solution.

In general, n-type doping processes of organic semiconductors are usually highly sensitive to traces of oxygen or water due to the formation of electron-rich doped intermediates^24^,^25^,^26^,^27^. To study the n-type doping process of the synthesized polymers and to evaluate the polymer charge carrier density by electrochemical doping, spectroelectrochemical measurements were carried out. The results are depicted in Fig. 4 where UV-Vis measurements are shown for a series of negative potentials applied to a thin film of p(gNDI-gT2).

As shown in Fig. 4, a new absorption feature can be observed between 550 and 650 nm when a negative potential is applied; the intensity of the feature increases incrementally on applying higher negative voltages. At higher doping levels (*V*=−0.7 V), two new absorption features within the ICT absorption band can be observed with a new absorption peak at 810 nm (*λ*_max_) and a broad band evolving at 900–1,100 nm, while the intensity of the initial ICT transition decreases correspondingly. Upon applying a negative potential during the spectroelectrochemical measurements, electrons are injected into the lowest unoccupied molecular orbital (LUMO) of the polymers to form a single occupied molecular orbital (SOMO) that enable new optical transitions similar to previously reported doping studies of P(NDI2OD-T2) (Polyera N2200)^28^,^29^. Consequently, the intensity of the charge transfer complex decreases while new absorption peaks arise from transitions between the HOMO and SOMO or SOMO and higher states.

Polymer p(gNDI-T2) shows a similar trend where the changes in absorption occur at slightly higher voltages, resulting from a lower EA of p(gNDI-T2) (Supplementary Fig. 6). It is likely that the n-type doping in aqueous solution only occurs to a certain degree before the polymers decompose, similar to PEDOT:PSS where over-oxidation degrades the material resulting in a lower conductivity^30^. For both polymers presented here, the resulting UV-Vis spectra are convolutions of un-doped and n-type doped polymer absorption bands, and a reversible n-type doping can be observed up to voltages of −0.5 V for p(gNDI-gT2) and −0.8 V for p(gNDI-T2) versus Ag/AgCl.

### Transistor characterization

**Figure 5:**
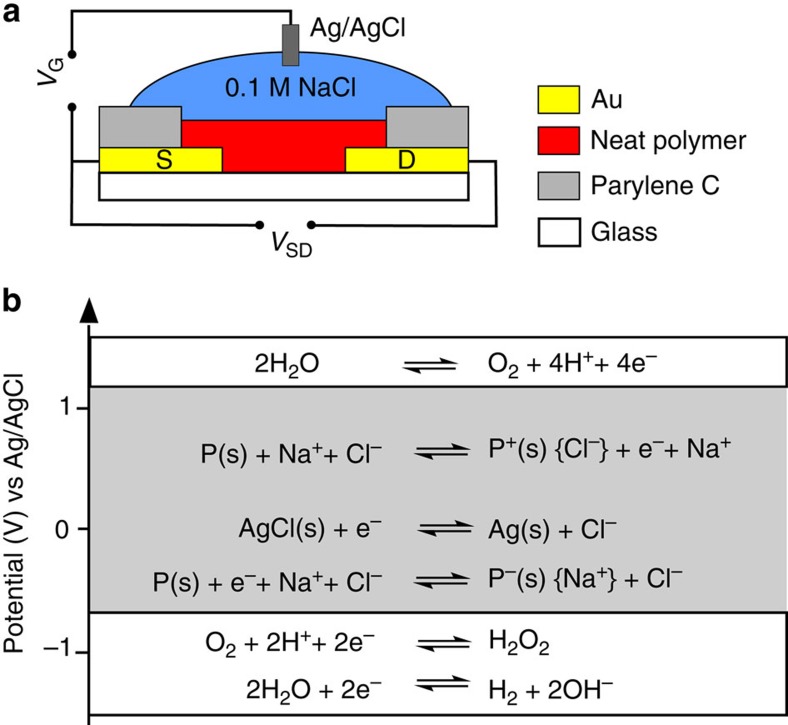
OECT structure and reactions governing operation. (**a**) Cross-sectional representation of the OECT architecture and (**b**) redox reactions of the polymer (P) in aqueous solutions. The OECT is operated in the common source configuration with a Ag/AgCl gate electrode. Electrochemical redox reactions of a polymer including the voltage limits of a stable operation in aqueous solution versus Ag/AgCl^26^,^31^.

The OECT is illustrated in Fig. 5a. Gold contacts (source and drain electrodes) and interconnects were patterned on a glass substrate where an additional layer of Parylene C insulates the gold interconnects. The conjugated polymer is deposited by spin coating without additives or further annealing steps and the device is operated in an aqueous 0.1 M NaCl solution with a Ag/AgCl gate electrode^6^,^8^. In accumulation mode, the electrochemical redox reactions are triggered by applying a positive (n-type doping) or negative (p-type doping) gate voltage. These reduction or oxidation reactions of the active material change the doping level of the semiconducting polymer and increase the conductivity of the polymer. Fig. 5b presents the limits of the gate potentials that can be applied versus Ag/AgCl for an OECT in water^31^.

**Figure 6:**
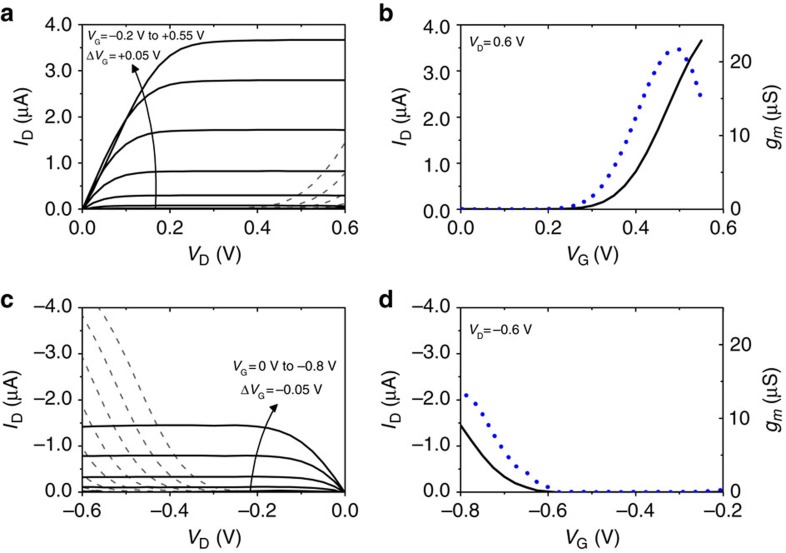
Performance of the ambipolar OECT. (**a**) n-type output characteristics, (**b**) n-type transfer curve (black) and transconductance (blue dotted), (**c**) p-type output characteristics, (**d**) p-type transfer curve (black) and transconductance (blue dotted) measured in 0.1 M NaCl aqueous solution. Output characteristics for n-type operation (**a**) are measured over the range of *V*_G_=−0.2 V to 0.55 V, with a *V*_G_ step size of 0.05 V (diode-like behaviour −0.2≤*V*_G_<0 is shown in grey (dashed)). For p-type operation (**c**), *V*_G_=0V to −0.8 V, with a *V*_G_ step size of 0.05 V (diode-like behaviour 0≥*V*_G_>−0.3 is shown in grey (dashed)). The device shown has channel dimensions of length 100 μm, width 10 μm and thickness 200 nm.

After demonstrating that the n-type doping and de-doping processes are fully reversible in aqueous solution, the performance of p(gNDI-T2) and p(gNDI-gT2)-based OECTs were measured. For polymer p(gNDI-gT2), it is possible to n-type dope the polymer with a channel length of 10 to 50 μm; the OECT performance is presented in Fig. 6a,b. Polymer p(gNDI-T2) on the other hand afforded currents that were below the measurement sensitivity for the given device geometry. Owing to its lower ionization potential, polymer p(gNDI-gT2) also performs as a p-type OECT (Fig. 6c,d) while the n-type device performance (current and transconductance) is found to be better than p-type operation. Table 2 summarizes the results of the transistor performance with two different device dimensions. Here, the best performing n-type device with a channel length of 10 μm and a width of 100 μm has a peak current of 3.85 μA (at *V*_G_=0.6 V) and a peak transconductance of 21.7 μS (at *V*_G_=0.5 V) with an ON/OFF ratio of 3.2 × 10^3^.

**Figure 7:**
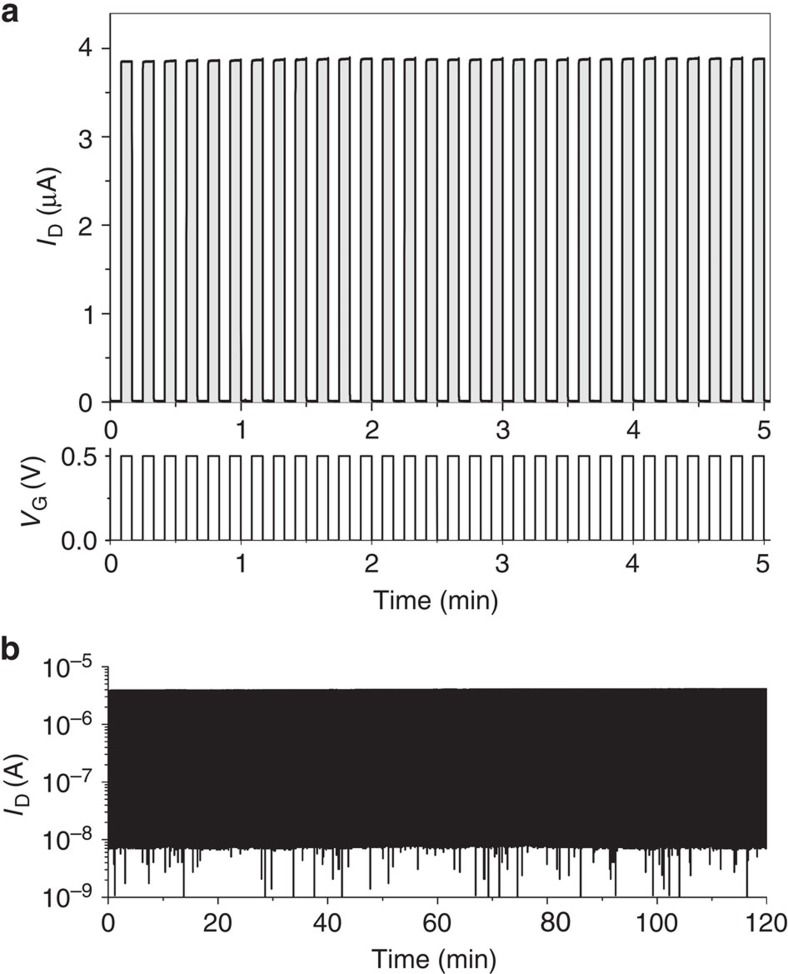
Long-term stability measurement of the transistor in 0.1 M NaCl aqueous solution. (**a**) Drain current and applied gate voltage pulses during the first 5 min of a 2 h operation, and (**b**) stable 2 h operation. A gate voltage pulse (*V*_G_=0.5 V) was applied for 5 s with an interval time of 5 s between the successive pulses (*V*_D_=0.5 V), the device shown has channel dimensions of length 100 μm, width 10 μm and thickness 350 nm. The response times of the device are ∼5 ms for both device turn on and turn off. Frequency-dependent transconductance is included in the Supplementary Information (Supplementary Fig. 12).

One of the major requirements for OECT recordings of biological processes^1^,^4^ is stable operation without degradation of the active material. Fig. 7a presents the stability of the current generated in the n-type OECT (*V*_D_=0.5 V) upon successive gate voltage pulses (Δ*V*_G_=0.5 V, pulse length=5 s) for five minutes. The device performance is extremely stable and only at gate voltages higher than *V*_G_=0.5 V, a slow device degradation can be observed, which is in agreement with the spectroelectrochemical stability measurements presented above. Long term stability measurements are presented in Fig. 7b where a 2 h stable device operation is demonstrated with no sign of degradation. To demonstrate the versatile applicability of the OECT, the electrolyte is changed to a phosphate-buffered saline solution and equivalent performance is observed compared to a 0.1 M NaCl aqueous solution (Supplementary Fig. 10).

When this device is compared to an accumulation mode p-type OECT based on a conjugated polyelectrolyte^10^, current and transconductance values of p(gNDI-gT2) are an order of magnitude lower. The conductivity (*σ*) is the product of the charge of an electron/hole *q*, charge carrier concentration *n*_*i*_ (*n*_*i*_=*n* (electron) or *p* (holes)) and the electronic mobility *μ* of the material. The reason for low conductivities could be either a limited increase in the charge carrier density or a low electron mobility. Since the CV and UV-Vis measurements showed an increase of the charge carrier density, we seek to compare electron mobilities by investigating OFETs with these polymers. OFETs were tested with bottom gate bottom contact architecture and the film casting/processing was kept the same as with OECTs, such that no additional OFET device optimization (contact treatments or annealing) was performed (Supplementary Figs 14 and 15). The electron mobilities (*μ*_*e*_) were measured to be 1.0 × 10^−4^ cm^−2^ V^−1^s^−1^ for p(gNDI-T2) and 1.0 × 10^−5^ cm^−2^ V^−1^s^−1^ for p(gNDI-gT2). Although charge transport occurs at the interface of the semiconductor and dielectric in a field effect transistor and in the bulk in an electrochemical transistor, we seek to compare the relative mobilities of the polymers and relate them to the device performance. We believe that the low electron mobility of p(gNDI-gT2) is the limiting factor to obtain high currents and transconductances as compared to previous accumulation mode OECTs^10^.

### X-ray scattering

**Figure 8:**
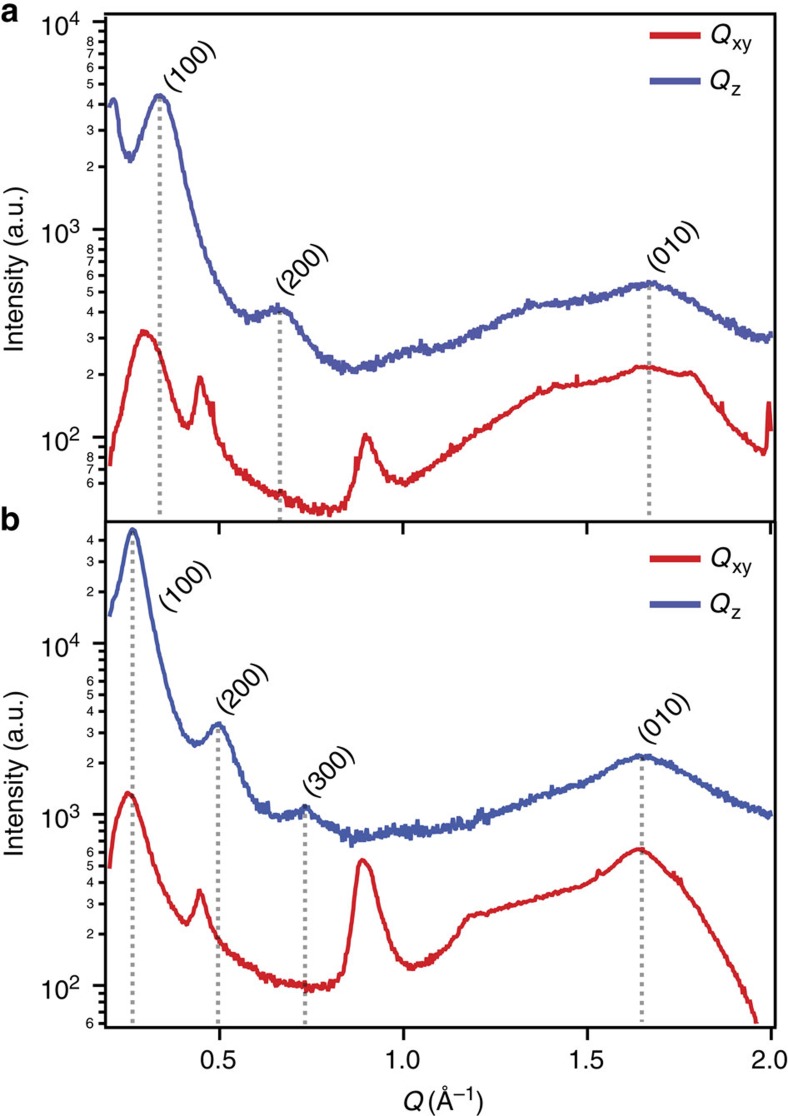
Two-dimensional grazing incidence X-ray scattering. 2D-GIXD line cuts of (**a**) p(gNDI-T2) and (**b**) p(gNDI-gT2). Cuts along the *Q*_xy_ direction (red) represent scattering in the plane of the substrate, while the scattering in the *Q*_z_ direction (blue) results from out-of-plane scattering. The associated lamellar (h00), and pi-stacking (0k0) peaks are indicated.

Grazing-incidence wide-angle X-ray scattering measurements of spin cast thin films reveal that both polymers show a propensity for the conjugated backbones to lie parallel to the substrate, with mixed lamellar/*π*-stacking texture. The general microstructure is evocative of the well-studied alkylated analogue p(NDI2OD-T2)^32^. In comparing p(gNDI-gT2) and p(gNDI-T2), the polymer with the higher glycol side chain density, p(gNDI-gT2), shows more readily observable higher order diffraction in the lamellar direction, and stronger scattering from the backbone and *π*-stacks as presented in Fig. 8. However, the crystallites/aggregates of p(gNDI-T2) show generally tighter packing in all directions, most notably in the lamellar stacking (*d*_lam_=2.07 nm [*q*_lam_=0.302 Å−^−1^] for p(gNDI-T2) versus *d*_lam_=2.38 nm [*q*_lam_=0.263 Å^−1^] for p(gNDI-gT2)) and *π*-stacking directions (*d*_*π*_=3.53 Å [*q*_*π*_=1.78 Å^−1^] for p(gNDI-T2) versus *d*_*π*_=3.73Å [*q*_*π*_=1.68 Å^−1^] for p(gNDI-gT2)). The reason for the closer packing of p(gNDI-T2) is most likely related to the lower glycol side chain density and may contribute to the higher FET mobility observed.

## Discussion

**Figure 9:**
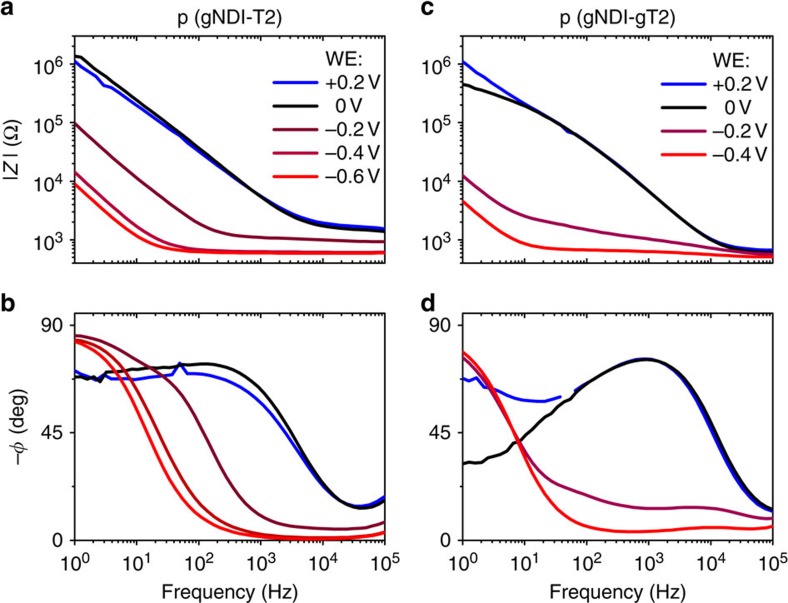
Electrochemical impedance spectroscopy. Bode plot and phase angle of p(gNDI-T2) with *d*=165 nm (**a**,**b**) and p(gNDI-gT2) with *d*=250 nm (**c**,**d**) in 0.1 M NaCl aqueous solution including the potentials applied at the working electrode (WE). Both polymers are cast on gold electrodes, and are 3.48 × 10^−3^ cm^−2^ in area.

A stable operation of an n-type electrolyte gated organic field effect transistor (EGOFET) was already reported where the operational voltage was reduced by changing from a solid state dielectric gated to an electrolyte gated device^33^. OECT performance, in comparison to EGOFET, relies on a large capacitance in the channel. From the measurements of electrochemical impedance spectroscopy of the polymers in 0.1 M NaCl aqueous solution (Fig. 9), it is possible to extract the effective capacitance per unit area. This is a quantitative means to determine whether doping of the polymer is largely electrostatic (and limited to the polymer/electrolyte interface—typical of EGOFET^34^) or whether the doping permeates the thickness of the channel, a defining characteristic of OECTs. A potential was applied to the polymer-coated gold electrode (working electrode (WE)) with respect to the Ag/AgCl reference electrode in 0.1 M NaCl aqueous solution, and the change in impedance was monitored. The effective capacitance per unit area of p(gNDI-gT2) is 9.9 × 10^−3^ F cm^−2^ at an offset potential of —0.4 V and is more than three orders of magnitude higher than for a P3HT-based EGOFET where a capacitance per area of 3–6 × 10^−5^ F cm^−2^ is measured on gold electrodes (and is relatively insensitive to the applied offset)^34^.

The device presented herein can therefore be categorized as a working OECT, due to the fact that (a) the turn on voltages of the OECT during n- and p-type operation are in agreement with the reduction and oxidation values obtained from CV measurements, (b) bulk electrical doping was verified by spectroelectrochemical measurements, and (c) the ability to store charge extends into the bulk of the film, yielding high capacitance values (with areal capacitances far greater than those of an EGOFET).

In comparing OECTs prepared with p(gNDI-gT2) to those with p(gNDI-T2), it is interesting to note that the material with the higher FET mobility (and tighter *π*-stacking) happens to be the material that did not work as an electrochemical transistor. Previous work has discussed the performance of PEDOT-based OECTs on the grounds of both electronic mobility, and capacity for charge due to electrochemical (de)doping upon ion injection^6^. The improved energy levels when comparing p(gNDI-gT2) to p(gNDI-T2), mainly a higher EA, allow for a decrease in the turn on voltage for polymer p(gNDI-gT2) and must lead to more facile cation injection/n-type doping. The enhanced ion injection is likely facilitated by higher glycol side chain density, and the resulting less densely packed conjugated backbones. Accounting for the thickness of the polymer layer, the estimated volumetric capacitance (C*) extracted from both the effective capacitance at 1 Hz as well as an equivalent circuit model fit to the impedance data at a bias of −0.4 V is 190 F cm^−3^ and 397 F cm^−3^ for p(gNDI-T2) and p(gNDI-gT2), respectively (model fits, Supplementary Fig. 13). Therefore, a combination of the doping energetics within the water-imposed stability window, and the higher capacity for electrochemical charge allow for p(gNDI-gT2) to operate as a stable, n-type (even ambipolar) accumulation mode OECT. In comparison to PEDOT:PSS, the volumetric capacitance of p(gNDI-gT2) is one order of magnitude higher^6^, demonstrating the potential for ethylene glycol chains to become the side chain of choice for OECT materials.

We demonstrate the successful development of an ambipolar OECT, opening a new direction for n-type conjugated polymers. This device was developed utilizing semiconducting copolymers comprising naphthalene-1,4,5,8-tetracarboxylic diimide and bithiophene units to form materials with hybridized energy levels and therefore high electron affinities and low ionization potentials. During the doping and de-doping processes, ions drift into and out of the active layer to compensate positive and negative charges on the polymer backbone. Glycol side chains show a strong tendency to interact with hydrated ions and water and therefore the polymers were functionalized with ethylene glycol-based side chains, which facilitated electrochemical switching between the reduced, neutral and oxidized states in aqueous solution. Spectroelectrochemical measurements were carried out to demonstrate high stability for p- and n-type doping in aqueous solution. A remarkably stable OECT operation was achieved, where the device was operated for 2 h without degradation.

## Methods

### Materials characterization

Column chromatography was carried out with silica gel for flash chromatography from VWR Scientific. Microwave experiments were performed in a Biotage Initiator V 2.3. ^1^H and ^13^C NMR spectra were recorded on a Bruker AV-400 spectrometer at 298 K and are reported in ppm relative to TMS. Deuterated solvents were purchased from Sigma Aldrich. UV-Vis absorption spectra were recorded on UV-1601 (1,100 nm) and UV-2600 (1,400 nm) UV-VIS Shimadzu UV-Vis spectrometers. Electrospray (ESI-TOF) mass spectrometry was performed with a Micromass LCT Premier. MALDI-TOF spectrometry was conducted in negative linear mode on a Micromass MALDImxTOF with trans-2-[3-(4-tert-butylphenyl)-2-methyl-2-propenylidene]-malononitrile (DCTB) as the matrix.

### Electrochemical characterization

Cyclic voltammograms were recorded using an Autolab PGSTAT101 with a standard three-electrode setup with ITO-coated glass slides as the working electrode, a platinum mesh counter electrode and a Ag/AgCl reference electrode calibrated against ferrocene (Fc/Fc^+^). The measurements were carried in an anhydrous, degassed 0.1 M tetrabutylammonium hexafluorophosphate TBAPF_6_ acetonitrile solution or a 0.1 M NaCl aqueous solution as the supporting electrolyte at a scan rate of 100 mV s^−1^. For the CV measurements in acetonitrile, the glassware was dried at 100 °C and the cell was purged with argon during the measurement to prevent oxygen contamination. Ionization potentials were obtained using the equation: IP=(*E*_ox_ – *E*_Fc_ + 4.8 V). Electrochemical impedance spectroscopy was performed with a three-electrode configuration using a potentiostat (Metrohm Autolab) with platinum and Ag/AgCl counter and reference electrodes, respectively. The polymer-coated gold electrode was the working electrode and the electrolyte was a 0.1 M NaCl aqueous solution. Effective capacitance was determined from C∼1/(2*πf*Im(Z)) where *f* is the frequency, and *Z* is the complex impedance; this capacitance was confirmed for doped spectra from a fit to a *R*(*R*||*C*) equivalent circuit in order to extract both capacitance per unit area, and C*. Analysis was performed with Metrohm NOVA software and custom MATLAB tools. Spectroelectrochemical measurements were performed using a PGSTAT101 potentiostat. ITO-coated glass slides with spun cast polymer was used as the working electrode immersed in a cuvette. A UV-1601 UV-VIS Shimadzu UV-Vis spectrometer was employed with the beam path passing through the cuvette filled with a 0.1 M NaCl aqueous solution and the polymer/ITO/glass sample. A background spectrum with the cuvette/electrolyte/ITO/glass was recorded for baseline correction before the experiment was started. Potential were applied for 5 s before the UV-Vis spectra were recorded.

### Transistor fabrication and characterization

OECTs were fabricated as previously reported^8^, except that the polymers p(gNDI-T2) and p(gNDI-gT2) were spun cast from chloroform before sacrificial peel off of Parylene C for the dry patterning processes. The completed samples were not annealed or treated after deposition, the samples were briefly rinsed in deionized water before testing. OECT IV curves (transfer and output), as well as repetitive pulsing were performed with a Keithley 2400 source-measure unit, and custom LabView scripts. Analysis was performed with MATLAB.

### Data availability

The authors declare that the data supporting the findings of this study are available within the paper and its supplementary information files.

## Additional information

**How to cite this article:** Giovannitti A. *et al*. N-type organic electrochemical transistors with stability in water. *Nat. Commun.* **7**, 13066 doi: 10.1038/ncomms13066 (2016).

## Supplementary Material

Supplementary Information Supplementary Figures 1-24, Supplementary Table 1, Supplementary Note 1, Supplementary Methods and Supplementary References

## Figures and Tables

**Table 1:** Properties of p(gNDI-T2) and p(gNDI-gT2).

**Polymer**	IP* **[eV]**	EA* **[eV]**	**Bandgap ΔE=IP−EA**	**Absorption onset [nm]**	**Repeating units [NMR]**
p(gNDI-T2)	5.53	3.85	1.68	817	—†
p(gNDI-gT2)	4.83	4.12	0.71	1,550	7‡

Ionization potential (IP), electron affinity (EA) and bandgap measured by cyclic voltammetry (CV) of a thin film in 0.1 M TBAPF_6_ (ACN) acetonitrile solution.

^*^CV measurements of the films with the onset energy value for oxidation and reduction peak.

^†^End capping for p(gNDI-T2) could not be carried out due to a low solubility of the polymer.

^‡^The number of repeating units was calculated by ^1^H NMR spectroscopy as well as MALDI-TOF spectrometry.

**Table 2:** OECT performance of p(gNDI-gT2).

**Device size (W, L) [μm]**	**Accumulation mode**	**peak currents [μA]**	**peak** ***g***_**m**_**(at |*****V***_**D**_|=0.6) [μS]*	***I***_**ON/OFF**_
100, 10	n-type	3.85	21.7	3.21 × 10^3^
	p-type	−1.45	13.4	2.01 × 10^2^
50, 50	n-type	0.47	2.72	5.41 × 10^2^
	p-type†	—	—	—

Thickness of the active layer is 200 nm.

^*^Gate voltage for peak *g*m (*V*_G_=0.5 V, (n-type) and (*V*_G_=−0.8 V, (p-type)).

^†^Low currents for the 50,50 device (p-type) lead to unreliable extraction of device parameters.
