# Erratum: N-type organic electrochemical transistors with stability in water

**DOI:** 10.1038/ncomms13955

**Published:** 2016-12-15

**Authors:** Alexander Giovannitti, Christian B. Nielsen, Dan-Tiberiu Sbircea, Sahika Inal, Mary Donahue, Muhammad R. Niazi, David A. Hanifi, Aram Amassian, George G. Malliaras, Jonathan Rivnay, Iain McCulloch

Nature Communications 7: Article number: 13066; DOI: 10.1038/ncomms13066 (2016); Published: 10 07 2016; Updated: 12 15 2016

Typographical errors were inadvertently introduced into the unit of charge mobility during the production process, such that the correct unit cm^2^ V^−1^ s^−1^ was incorrectly give as cm^−2^ V^−1^ s^−1^. These errors do not affect the analysis of the results presented in the Article.

The penultimate sentence of the second paragraph of the Article should read ‘Recently, the electron mobility of n-type polymers has increased rapidly, reaching values of more than 1.0 cm^2^ V^−1^ s^−1^ in OFETs,^12–17^ thus enabling n-type OECTs operating in accumulation mode.'

Similarly, the final sentence of the fourth paragraph of the section ‘Transistor characterization' should read ‘The electron mobilities (μ_e_) were measured to be 1.0 × 10^−4^ cm^2^ V^−1^ s^−1^ for p(gNDI-T2) and 1.0 × 10^−5^ cm^2^ V^−1^ s^−1^ for p(gNDI-gT2).'

