# Supplementary material for: N-type organic electrochemical transistors with stability in water
Source: Nat Commun. 2016 Oct 7;7:13066. doi: 10.1038/ncomms13066 (PMC5059848; doi:10.1038/ncomms13066)
Supplement: Supplementary Information — Supplementary Figures 1-24, Supplementary Table 1, Supplementary Note 1, Supplementary Methods and Supplementary References [file ncomms13066-s1.pdf]

Supplementary Figures

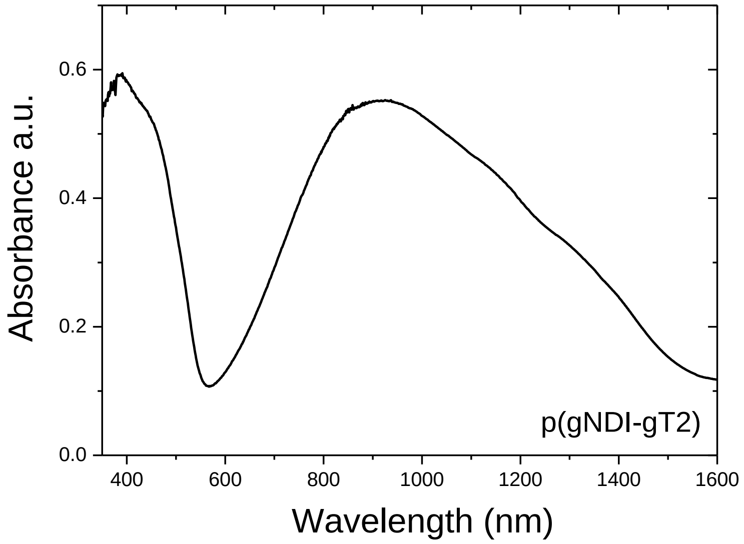

Supplementary Figure 1: UV-Vis spectrum of p(gNDI-gT2) in solid state using a NIR detector.

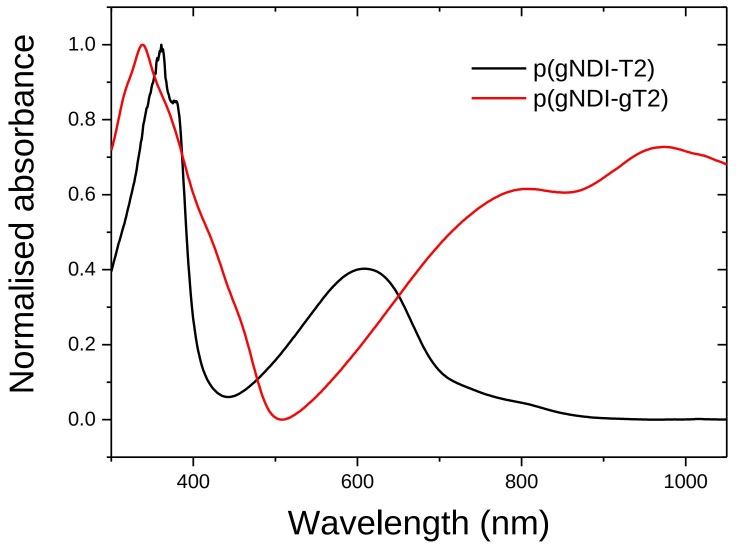

Supplementary Figure 2: UV-Vis spectrum of p(gNDI-T2) and p(gNDI-gT2) in chloroform.

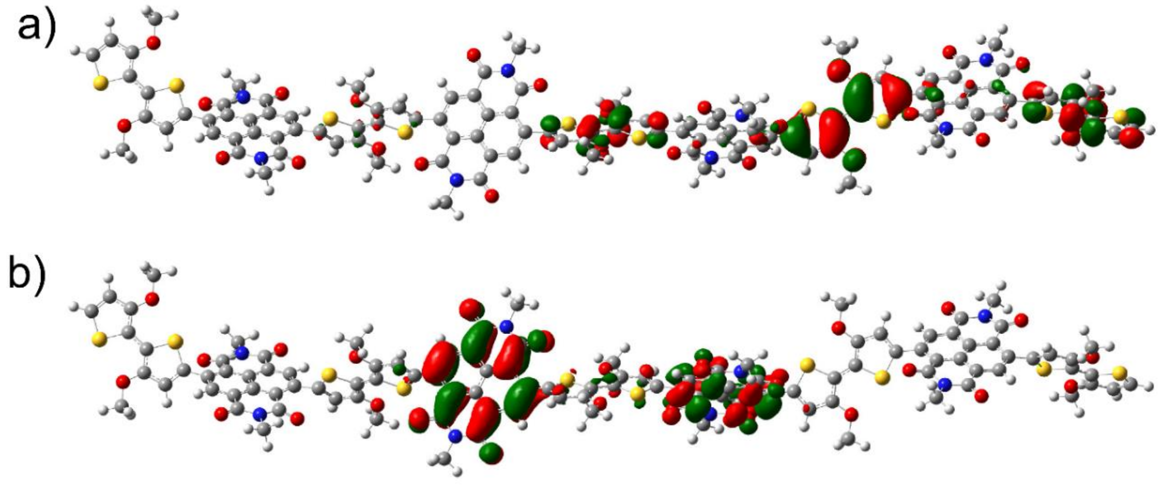

Supplementary Figure 3: DFT calculations of p(gNDI-gT2), (a) HOMO and (b) LUMO at WB97XD/6-31G(d,p) level of theory.

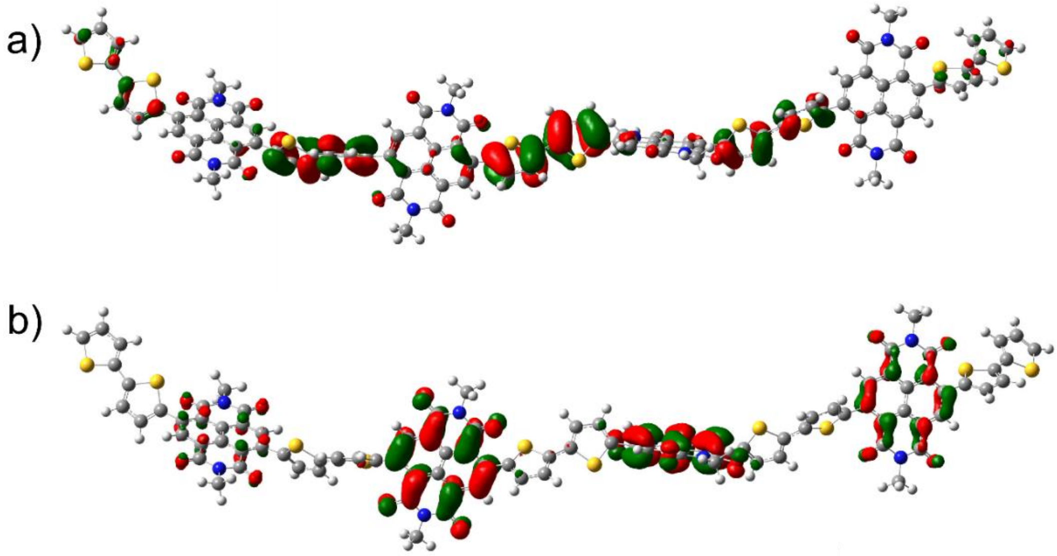

Supplementary Figure 4: DFT calculations of p(gNDI-T2), (a) HOMO and (b) LUMO at WB97XD/6-31G(d,p) level of theory.

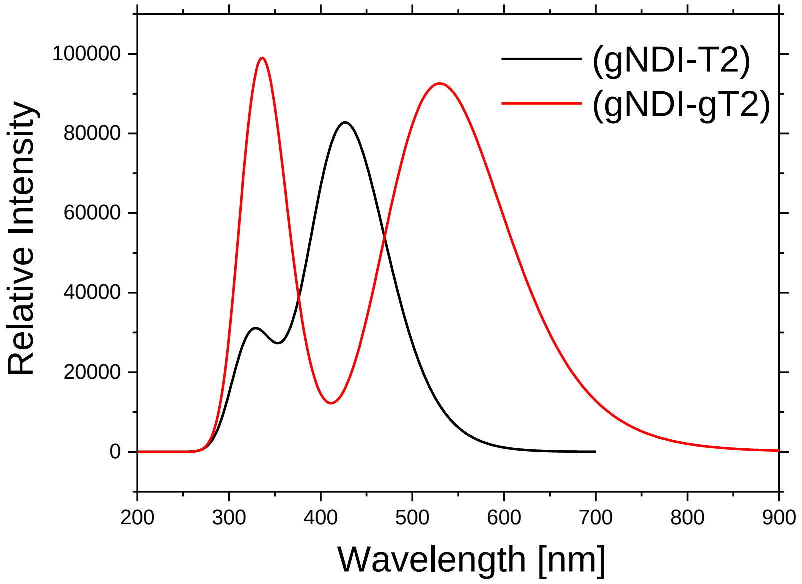

Supplementary Figure 5: Time dependent (TD) DFT calculations: Simulated UV-Vis spectrum of a ABABABABA molecule where A is either T2 or gT2 and B is gNDI at WB97XD/6-31G(d,p) level of theory.

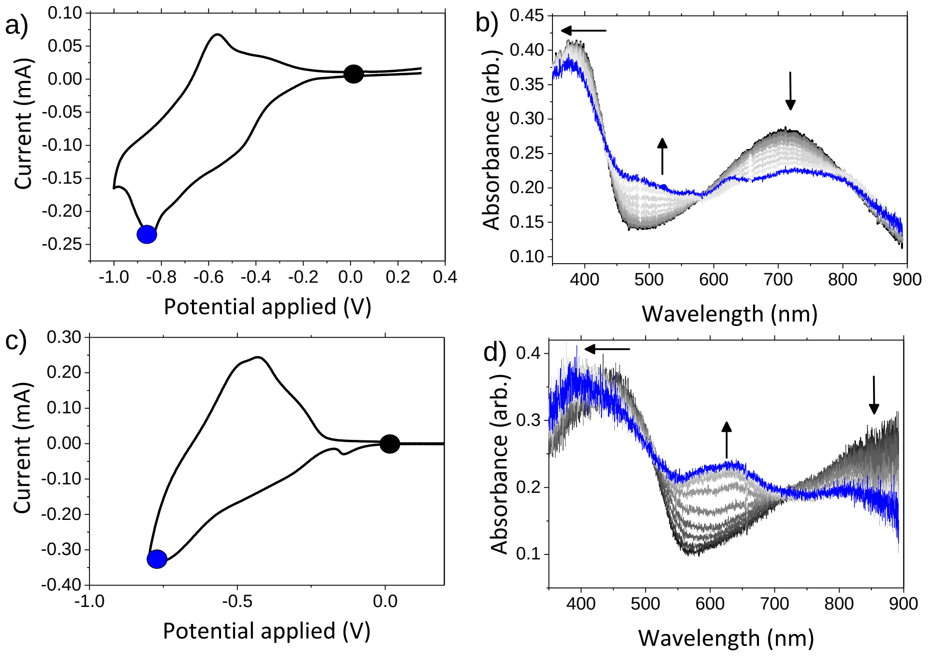

Supplementary Figure 6: Spectroelectrochemical measurements of p(gNDI-T2) (a, b) and p(gNDI-gT2) (c,d) (n-type doping).

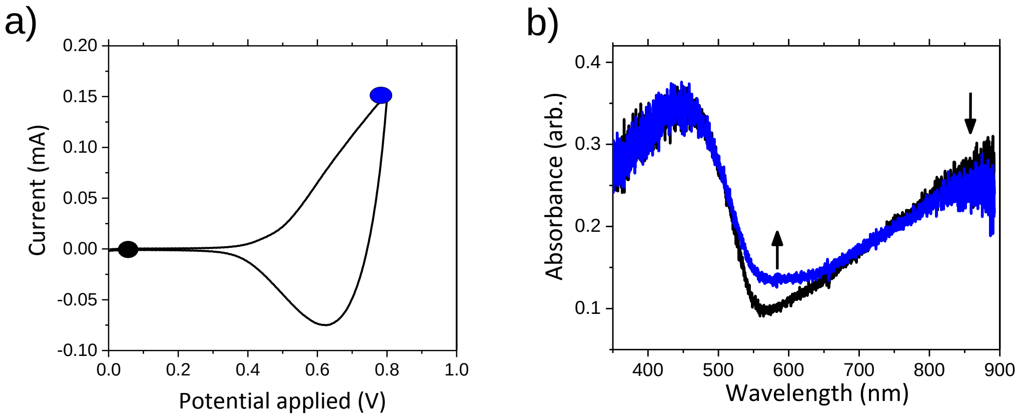

Supplementary Figure 7: Spectroelectrochemical measurements of p(gNDI-gT2) (a, b) (p-type doping).

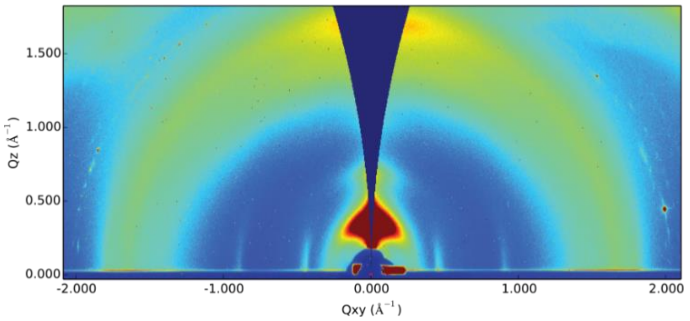

Supplementary Figure 8: Two-dimensional grazing incidence X-ray diffraction of p(gNDI-T2).

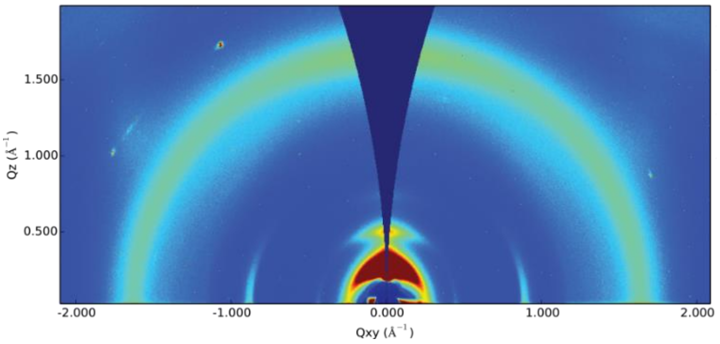

Supplementary Figure 9: Two-dimensional grazing incidence X-ray diffraction of p(gNDI-gT2).

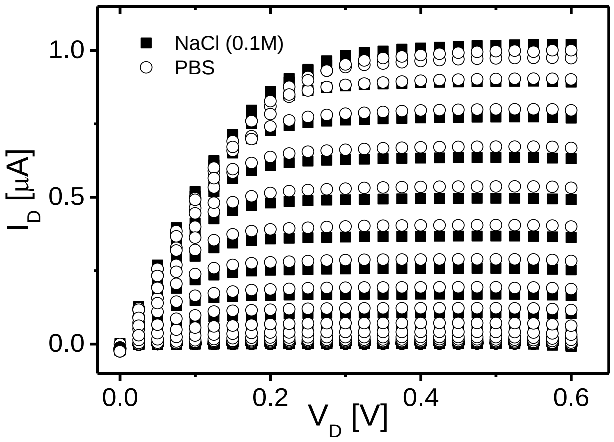

Supplementary Figure 10: OECT measurements were carried out in 0.1 M NaCl solution and PBS solution (V_G = 0 to 0.6 V, V_D = 0.6 V).

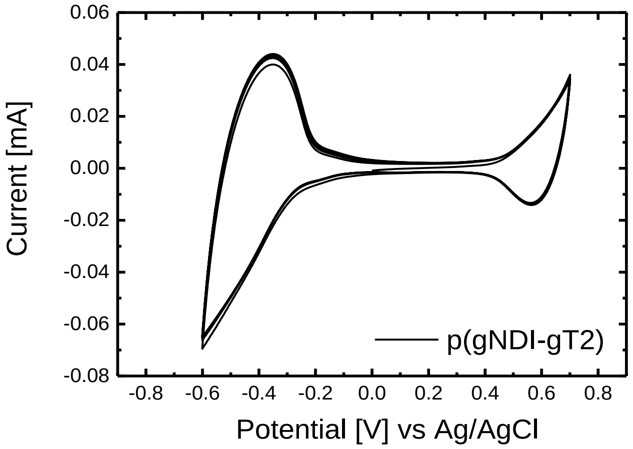

Supplementary Figure 11: CV measurements on ITO coated glass substrates of p(gNDI-gT2) in PBS solution (10 cycles, 100 mV/s).

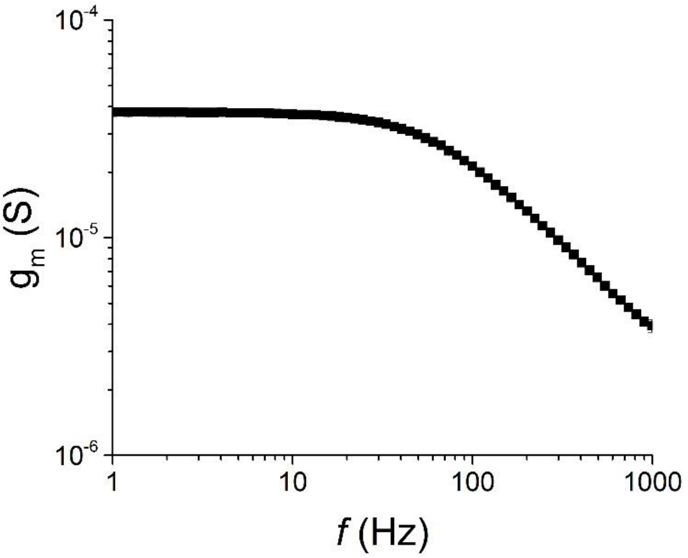

Supplementary Figure 12: Frequency dependent transconductance (W = 100, L = 10, d = ~350 nm). The drain current is kept constant at $V_d = 0.5V$, while a sine wave of $\Delta V_g = 10mV$ amplitude and $V_{g,offset} = 0.5V$ (varying frequencies) is applied. The frequency dependent transconductance is determined from the resulting sine wave in the drain current, $g_m = \Delta I_d / \Delta V_g$.

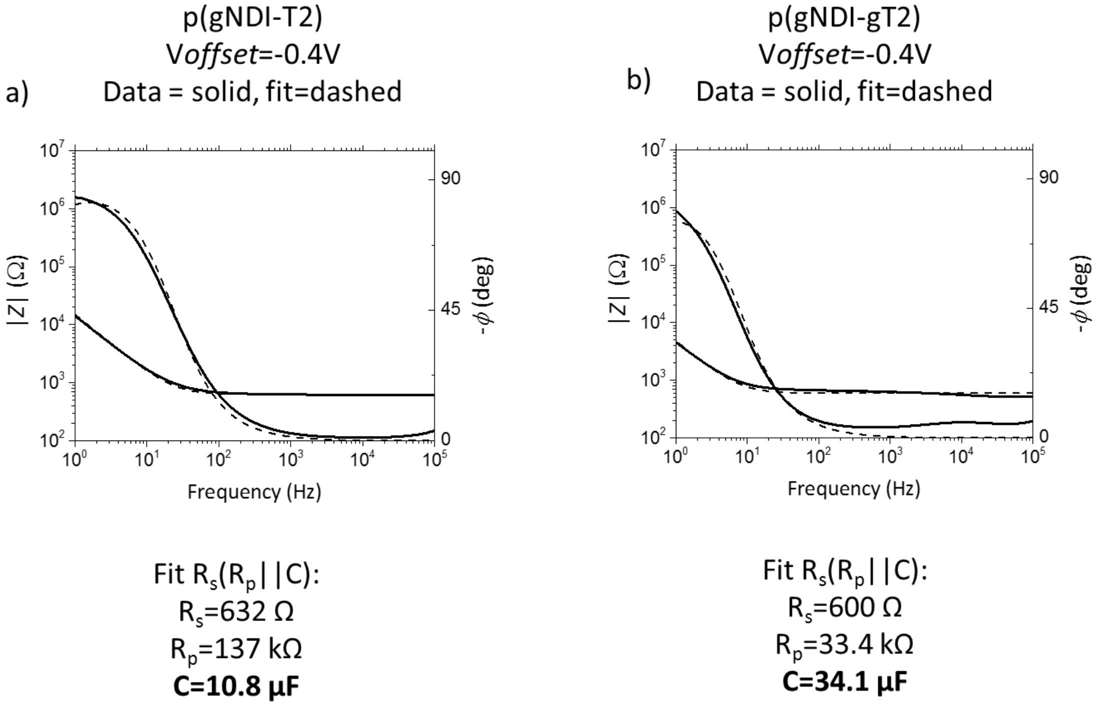

Supplementary Figure 13: Electrical impedance spectroscopy of (a) p(gNDI-T2) and (b) p(gNDI-gT2) at an offset bias of V = −0.4V with the fits for a $R_s(R_P \text{ II } C)$ circuit.

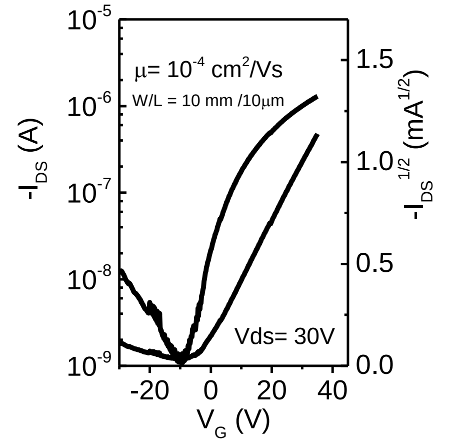

Supplementary Figure 14: Transfer curve p(gNDI-T2).

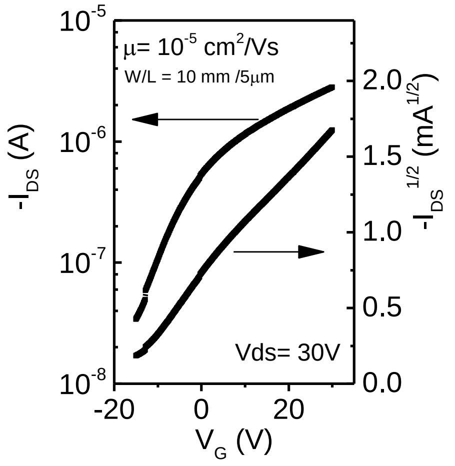

Supplementary Figure 15: Transfer curve p(gNDI-gT2).

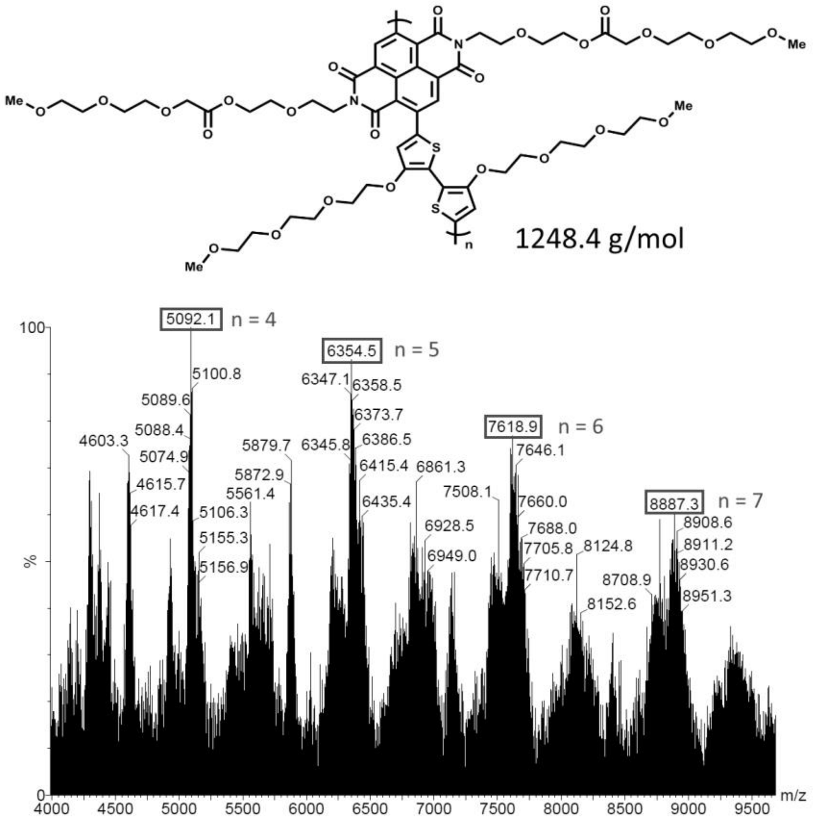

Supplementary Figure 16: Mass spectrum of p(gNDI-gT2).

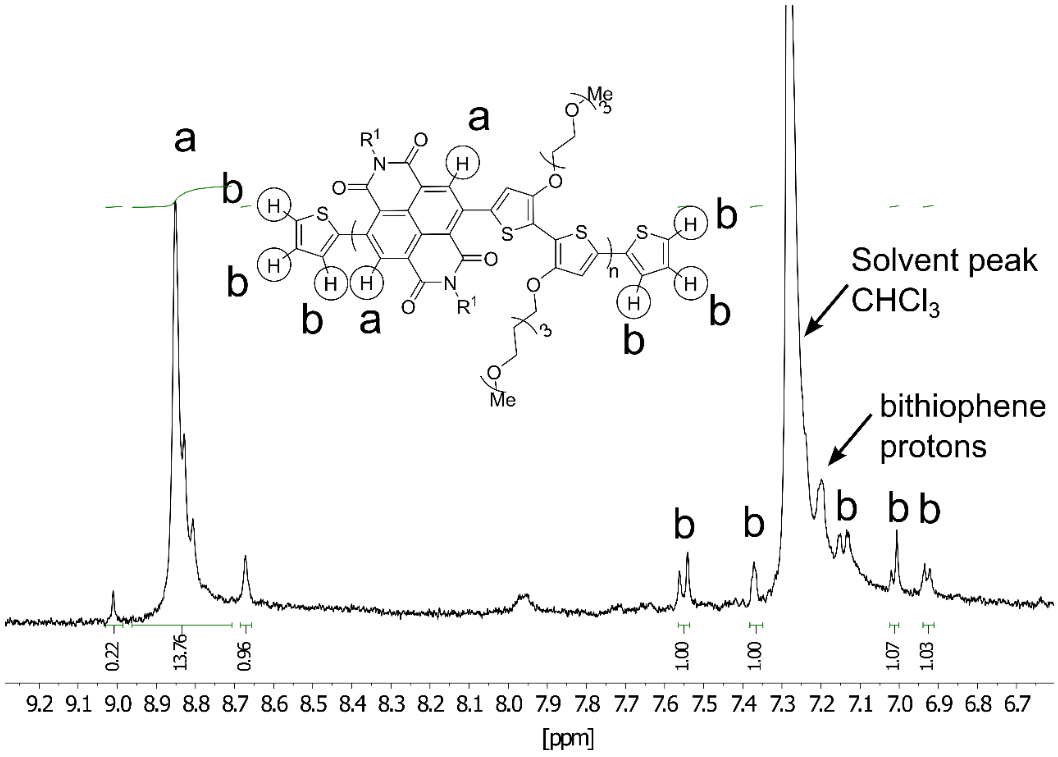

Supplementary Figure 17: ^{1}H NMR of p(gNDI-gT2) in the region of 9.2 - 6.7 ppm.

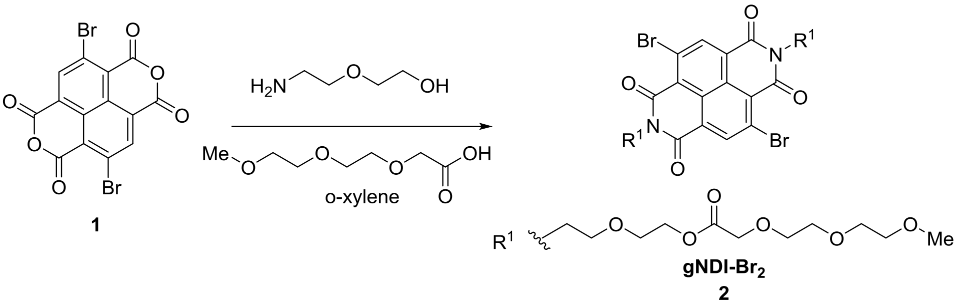

Supplementary Figure 18: Synthesis of gNDI-Br_2.

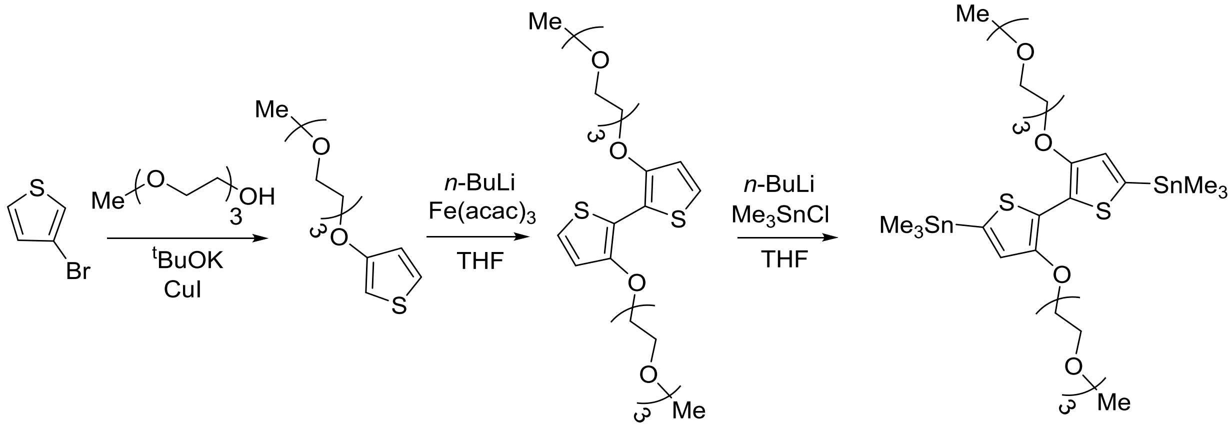

Supplementary Figure 19: Synthesis of (3,3'-bis(2-(2-(2-methoxyethoxy)ethoxy)ethoxy)-[2,2'-bithiophene]-5,5'-diyl)bis(trimethylstannane) according to the literature.[2]

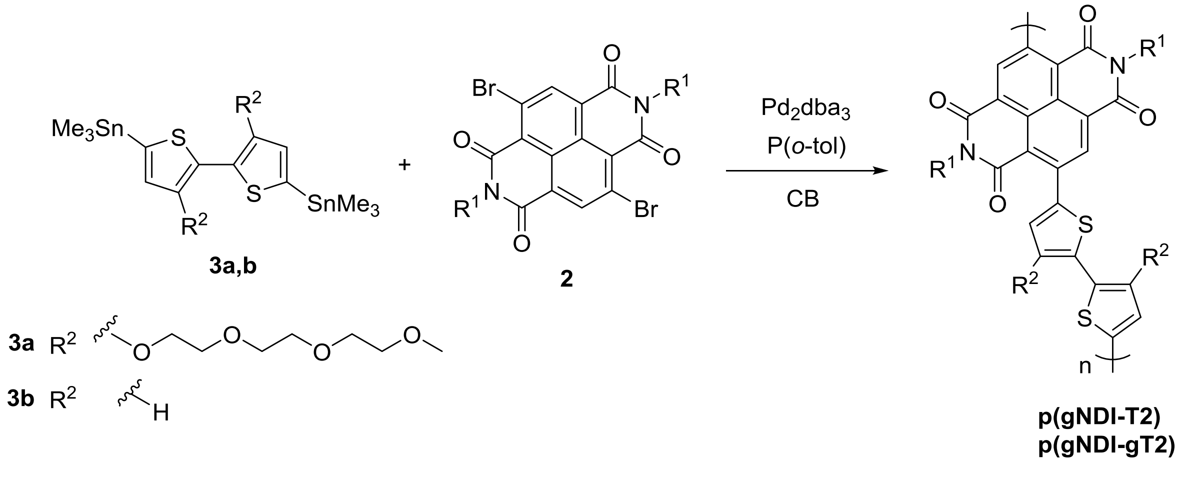

Supplementary Figure 20: Stille Polymerisation

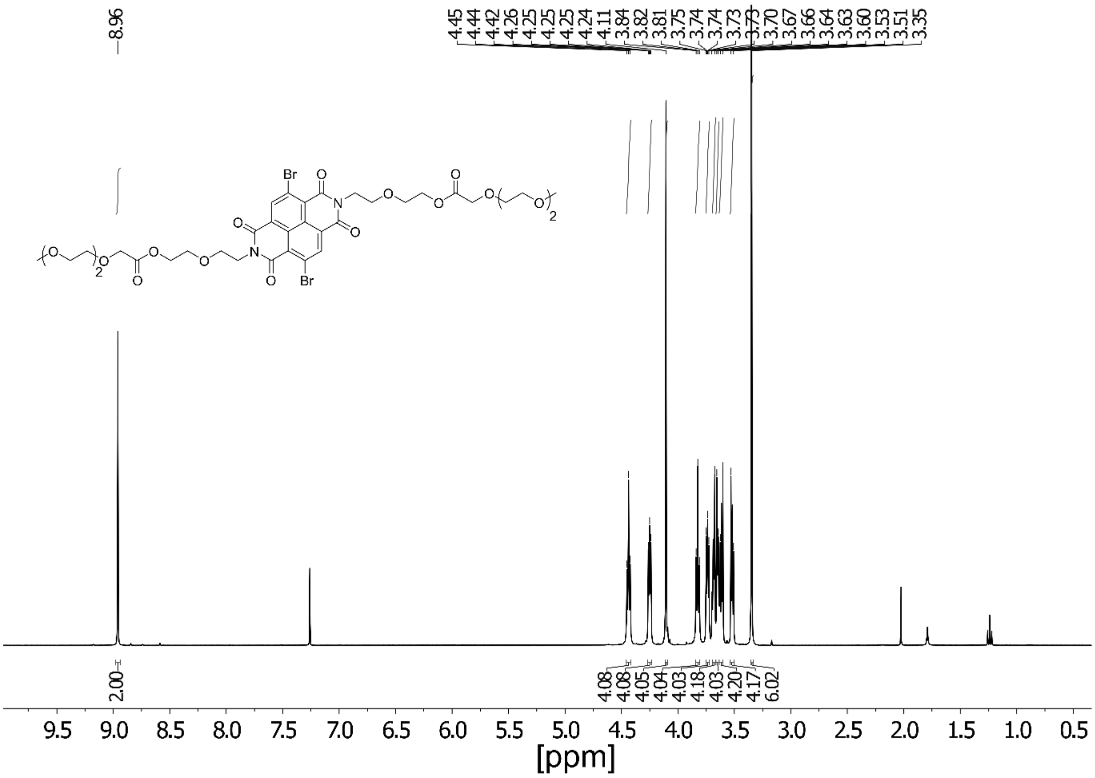

Supplementary Figure 21: ^{1}H NMR of gNDI-Br_2 in $CDCl_3$.

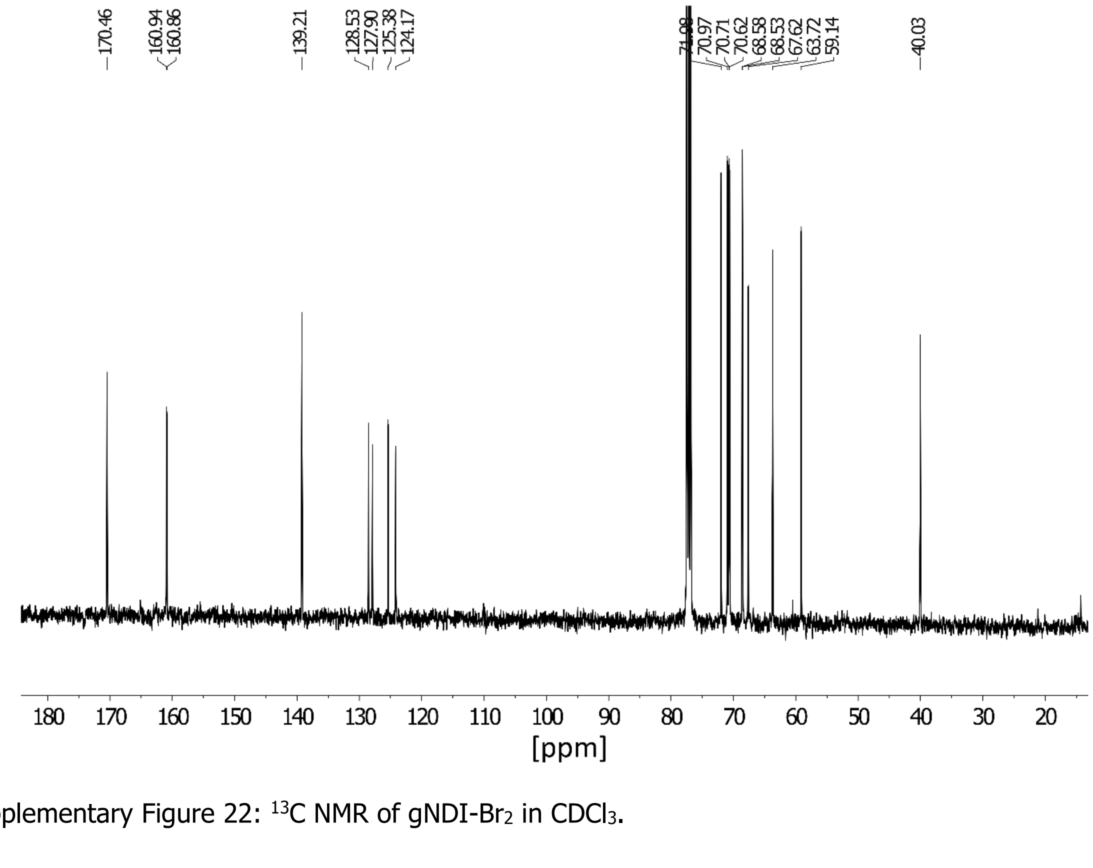

Supplementary Figure 22: ^{13}C NMR of gNDI-Br_2 in $CDCl_3$.

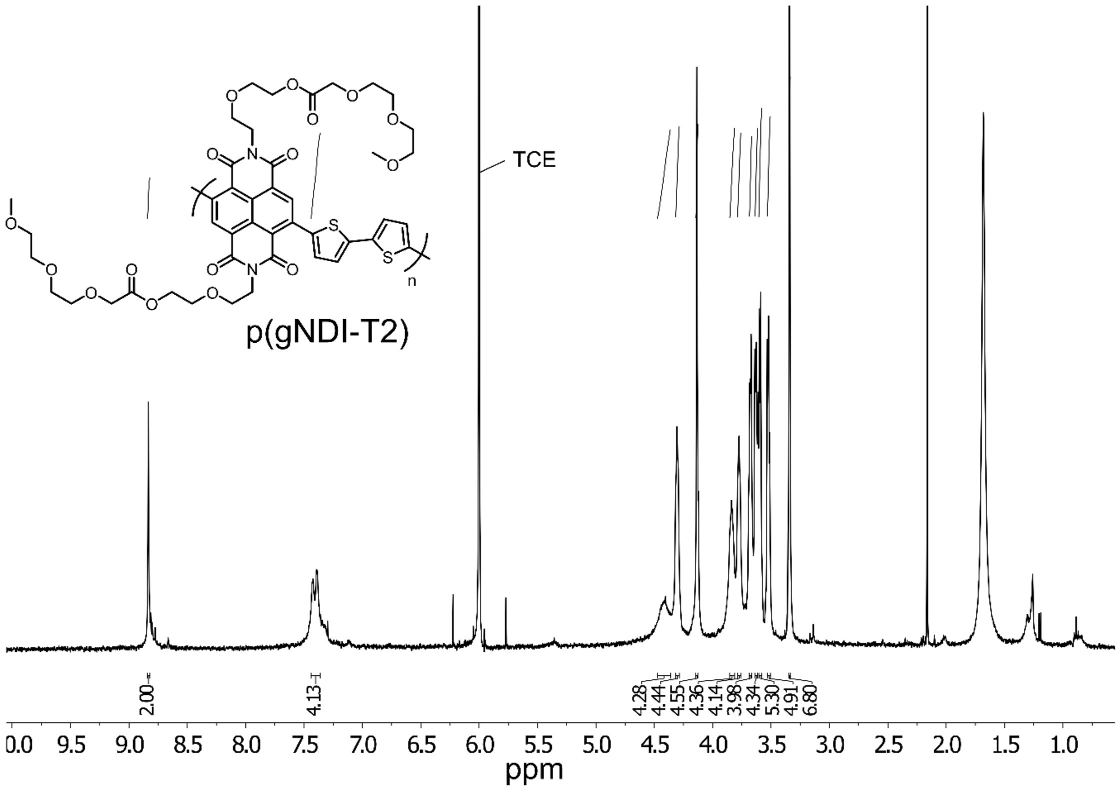

Supplementary Figure 23: ^{1}H NMR of p(gNDI-T2) in TCE-d_2.

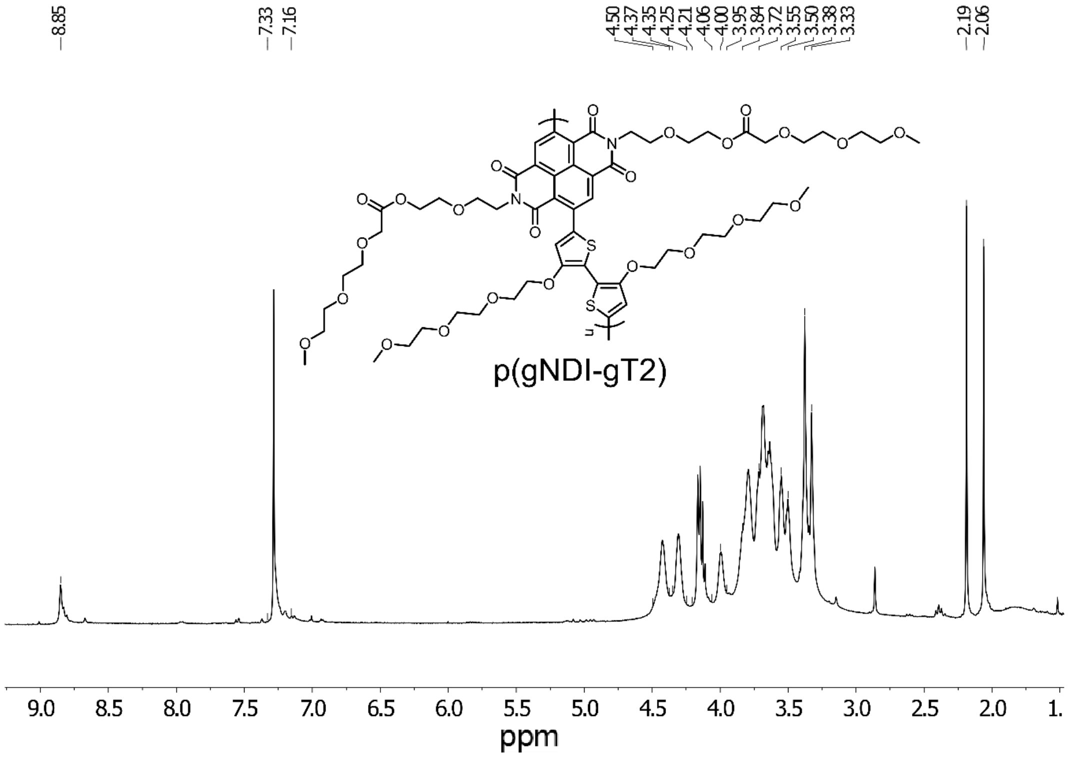

Supplementary Figure 24: ^{1}H NMR of p(gNDI-gT2) in $CDCl_3$.

Supplementary Table

Supplementary Table 1: Summary OFET measurements.

Polymer	μ_e avg. [cm²/Vs]	W/L
**p(gNDI-T2)**	$1.0*10^{-4}$	10 mm/ 10 µm
**p(gNDI-gT2)**	$1.0*10^{-5}$	10mm/ 5 µm

Supplementary Note 1

Molecular weight distribution. The amount of repeating units of p(gNDI-gT2) was measured by mass spectrometry and ^{1}H NMR spectroscopy. MALDI TOF measurements were conducted with DCTB as the matrix and repeating units up to 7 units were detected as shown in Supplementary Figure 16. For the ^{1}H NMR measurements presented in Supplementary Figure 17, the ratio of the thiophene protons b of the end-capping units were related to the NDI protons a (the ratio of the NDI unit to the end group thiophenes is 2: 1, Supplementary Figure 17) The proton ratio of a:b is 14:1 and the average chain length can be calculated to be seven repeating units. End capping for p(gNDI-T2) could not be carried out due to a low solubility of the polymer. The high polarity of the polymers did not allow the molecular weight analysis by gel permeation chromatography (GPC) in solvents as e.g. chlorobenzene, chloroform or DMF.

Supplementary Methods

Materials. 2,6-Dibromonaphthalene-1,4,5,8-tetracarboxylic dianhydride was purchased from TCI. [2-(2-Methoxyethoxy)ethoxy]acetic acid, tris(dibenzylideneacetone)-dipalladium(0) ($Pd_2(dba)_3$), tri(*o*-tolyl)phosphine were purchased from VWR. 2-(2-Aminoethoxy)ethanol, anhydrous xylene and chlorobenzene were purchased from Sigma Aldrich.

Electrical characterisation. Spectroelectrochemical measurements with fiber/white light spectrometer (Supplementary Figure 6 and 8) were performed using a PGSTAT101 potentiostat. An ITO-coated glass slide with spun cast polymer was used as the working electrode immersed in a cuvette. A fiber/white light spectrometer with white light source (Hamamatsu) and Ocean Optics USB4000 spectrometer were employed with the beam path passing through the filled cuvette and polymer/ITO/glass sample. A background spectrum with cuvette/electrolyte/ITO/glass was recorded before the experiments were carried out. The absorption was measured in 1s intervals as the CV experiment was performed.

Bottom-gate, bottom-contact (BGBC) organic thin film transistors (OTFTs) were fabricated on Si/SiO_2 substrate with a 240nm SiO_2 layer as the dielectric and gold electrodes of 45 nm thickness with 5 nm ITO as adhesion layer for Au contacts with SiO_2. Devices had channel lengths and widths of 5-20 μm and 10 mm, respectively. Polymer p(gNDI-gT2) was dissolved in chloroform and p(gNDI-T2) in 1,1,2,2-tetrachloroethane with a concentration of 5 mg/ml. The polymer solutions were stirred overnight. The solutions were spin coated at 1000 rpm for 60 seconds. Electrical measurements were performed in a nitrogen atmosphere with a semiconductor parameter analyser (Kiethley 4200 SCS). Field effect mobilities were calculated using the standard thin film model in saturation regime of the device $\mu_{sat} = \frac{2L}{WC} (\frac{\partial I_{dsat}}{\partial V_g})^2$, where L, W and C are the channel length, channel width and geometric capacitance of the dielectric, respectively. The same processing conditions were used for OFETs and for OECTs in order to achieve similar film forming properties. No additional device optimisation (contact treatment and annealing) was carried out.

DFT calculations. Density functional theory (DFT) calculations of a molecule with the repeating units ABABABABA (A is gT2 or T2, B is gNDI) were carried in the gas phase at WB97XD/6-31G(d,p) level of theory. Calculations were carried out with short methoxy side chains to lower the computational time.

X-ray scattering. Samples for X-ray scattering were coated on native oxide, silicon wafer pieces, in the same manner as for device measurements. Wide angle GIXD was performed at the Stanford Synchrotron radiation lightsource (SSRL) at beamline 11-3 with a photon energy of 12.73keV at a working distance of 326.84mm. The incidence angle α of the incident beam was set to 0.1°, above the critical thickness of the semiconducting film but below that of the silicon substrate. The diffraction intensity was detected on a 2D CCD area detector (Rayonix MAR-225). The distance between sample and detector was calibrated using a LaB6 polycrystalline standard. Sample detector distance calibration and 2D data reduction were performed using a combination of WxDiff and GIXSGUI software packages.[1]

Synthesis. gNDI-Br$_2$: 2,6-Dibromonaphthalene-1,4,5,8-tetracarboxylic dianhydride (765 mg, 1.8 mmol), 2-(2-aminoethoxy)ethanol (0.4 mL, 4.0 mmol, 2.2 eq.) were charged into a dried 50 mL two neck RBF under argon. 5.0 mL of [2-(2-methoxyethoxy)ethoxy]acetic acid and 20 mL of *o*-xylene were added and the reaction mixture was heated to 140 °C for 16 h. The reaction mixture was cooled to r.t. and the solvent was concentrated under reduced pressure. 50 mL of $CHCl_3$ was added and the organic phase was washed with water (3 x 50 mL). The organic phase was dried over $MgSO_4$ and the solvent was removed in *vacuo*. Column chromatography on silica gel was carried out with ethyl acetate/methanol (98:2). The solvent was removed and the yellow solid was recrystallised from ethyl acetate. 220 mg (0.24 mmol) of a yellow solid was obtained with a yield of 13 %. ^{1}H NMR (400 MHz, $CDCl_3$): 8.96 (s, 2H), 4.45 – 4.42 (t, *J* = 5.8 Hz, 4H), 4.26 – 4.24 (m, 4H), 4.11 (s, 4H), 3.84 – 3.81 (t, *J* = 5.7 Hz, 4H), 3.75 – 3.73 (m, 4H), 3.70 – 3.67 (m, 4H), 3.66 – 3.64 (m, 4H), 3.63 – 3.60 (m, 4H), 3.53 – 3.51 (m, 4H), 3.35 (s, 6 H) ppm. ^{13}C NMR (100 MHz, $CDCl_3$): 170.5, 160.9, 160.8, 139.2, 128.5, 127.9, 125.4, 124.2, 72.0, 71.0, 70.7, 70.6, 68.6, 68.5, 67.6, 63.7, 59.1, 40.0 ppm. HRMS (ES-ToF): 919.1120 [M-H$^+$] (calc. 919.1138), 936.1444 [M-H_3O^+] (calc. 936.1163).

p(gNDI-T2): In a 2.0 mL microwave vial, gNDI-Br$_2$ **2** (76.2 mg, 83.0 µmol) and 5,5′-bis(trimethylstannyl)-2,2′-bithiophene **3b** (40.7 mg, 83.0 µmol) were dissolved in 0.8 mL of anhydrous, degassed chlorobenzene. $Pd_2(dba)_3$ (1.5 mg, 1.7 µmol) and P(*o*-tol)$_3$ (2.0 mg, 6.8 µmol) were added and the vial was sealed and heated to 100 °C for 5 min, 120 °C for 5 min, 140 °C for 5 min, 160 °C for 5 min, 180 °C for 5 min and 200 °C for 25 min. After cooling to room temperature, the solid was collected by filtration into a thimble and soxhlet extraction was carried out with hexane, MeOH, acetone, THF and chloroform. A small fraction (5 mg) was soluble in chloroform while the main part of the polymer was only soluble in hot 1,1,2,2-Tetrachloroethane (TCE). The p(gNDI-T2) was obtained as a dark blue solid with a yield of 41 % (32.0 mg, 34 µmol). Due to the low solubility of the polymer in chlorobenzene it was not possible to perform end-capping. ^{1}H NMR (TCE-*d*$_2$, 400 MHz) δ: 8.83 (s, 2 H), 7.43-7.38 (m, 4H), 4.48-4.35 (m, 4H), 4.32-4.30 (m, 4H), 4.14 (s, 4H), 3.88-3.84 (m, 4H), 3.80-3.74 (m, 4H), 3.71-3.69 (m,4H), 3.64-3.62 (m, 4H), 3.61-3.59 (m, 4H), 3.53-3.51 (m, 4H), 3.34 (s, 6H) ppm.

p(gNDI-gT2): In a 2.0 mL microwave vial, gNDI-Br_2 **2** (46.2 mg 50.0 µmol) and (3,3'-bis(2-(2-(2-methoxyethoxy)ethoxy)ethoxy)-[2,2'-bithiophene]-5,5'-diyl)bis-(trimethylstannane) (40.9 mg, 50.0 µmol) were dissolved in 1.0 mL of anhydrous, degassed chlorobenzene. $Pd_2(dba)_3$ (0.8 mg, 1.0 µmol) and P(*o*-tol)$_3$ (1.2 mg, 4.0 µmol) were added and the vial was heated to 100 °C for 5 min, 120 °C for 5 min, 140 °C for 5 min, 160 °C for 5 min, 180 °C for 5 min and 200 °C for 25 min. After the reaction was cooled to room temperature, 0.1 mL of a solution made of 0.1 mL 2-(Tributylstannyl)thiophene in 0.5 mL of chlorobenzene and 0.75 mg of $Pd_2(dba)_3$ was added and heated for 5 min to 180 °C. After cooling to room temperature, 0.1 mL of a solution made of 0.1 mL 2-bromothiophene in 0.5 mL of chlorobenzene was added and heated to 180 °C. The reaction mixture was cooled to room temperature and the dark green reaction mixture was precipitated in hexane and the solids were collected by filtration into a thimble. Soxhlet extraction was carried out with hexane, MeOH, acetone, THF and chloroform. The polymer was dissolved in chloroform. Polymer p(gNDI-gT2) was obtained as a green solid with a yield of 21 % (13.0 mg, 0.01 mmol).

Supplementary References

1. Jiang, Z. GIXSGUI : a MATLAB toolbox for grazing-incidence X-ray scattering data visualization and reduction, and indexing of buried three-dimensional periodic nanostructured films. *J. Appl. Crystallogr.* **48,** 917–926 (2015).
2. Song, C. K., Eckstein, B. J., Tam, T. L. D., Trahey, L. & Marks, T. J. Conjugated Polymer Energy Level Shifts in Lithium-Ion Battery Electrolytes. *ACS Appl. Mater. Interfaces* **6,** 19347–19354 (2014).
